# Differential contribution of TRPM4 and TRPM5 nonselective cation channels to the slow afterdepolarization in mouse prefrontal cortex neurons

**DOI:** 10.3389/fncel.2014.00267

**Published:** 2014-09-04

**Authors:** Ya-Ting Lei, Sebastien J. Thuault, Pierre Launay, Robert F. Margolskee, Eric R. Kandel, Steven A. Siegelbaum

**Affiliations:** ^1^Department of Neuroscience, Columbia University Medical Center, New York State Psychiatric InstituteNew York, NY, USA; ^2^Equipe Avenir, Institut National de la Santé et de la Recherche Médicale, Service de Néphrologie, Hôpital Bichat, Université ParisParis, France; ^3^Monell Chemical Senses CenterPhiladelphia, PA, USA; ^4^Howard Hughes Medical Institute, Columbia UniversityNew York, NY, USA; ^5^Kavli Institute for Brain Sciences, Columbia UniversityNew York, NY, USA; ^6^Department of Psychiatry, Columbia UniversityNew York, NY, USA; ^7^Department of Pharmacology, Columbia UniversityNew York, NY, USA

**Keywords:** slow afterdepolarization (sADP), persistent firing, muscarinic receptors, Ca^2+^-activated non-selective cation (CAN) current, transient receptor potential melastatin 5 channel (TRPM5)

## Abstract

In certain neurons from different brain regions, a brief burst of action potentials can activate a slow afterdepolarization (sADP) in the presence of muscarinic acetylcholine receptor agonists. The sADP, if suprathreshold, can contribute to persistent non-accommodating firing in some of these neurons. Previous studies have characterized a Ca^2+^-activated non-selective cation (CAN) current (*I_CAN_*) that is thought to underlie the sADP. *I_CAN_* depends on muscarinic receptor stimulation and exhibits a dependence on neuronal activity, membrane depolarization and Ca^2+^-influx similar to that observed for the sADP. Despite the widespread occurrence of sADPs in neurons throughout the brain, the molecular identity of the ion channels underlying these events, as well as *I_CAN_*, remains uncertain. Here we used a combination of genetic, pharmacological and electrophysiological approaches to characterize the molecular mechanisms underlying the muscarinic receptor-dependent sADP in layer 5 pyramidal neurons of mouse prefrontal cortex. First, we confirmed that in the presence of the cholinergic agonist carbachol a brief burst of action potentials triggers a prominent sADP in these neurons. Second, we confirmed that this sADP requires activation of a PLC signaling cascade and intracellular calcium signaling. Third, we obtained direct evidence that the transient receptor potential (TRP) melastatin 5 channel (TRPM5), which is thought to function as a CAN channel in non-neural cells, contributes importantly to the sADP in the layer 5 neurons. In contrast, the closely related TRPM4 channel may play only a minor role in the sADP.

## Introduction

Cholinergic receptor activation is important for various memory processes (Spencer et al., 1985; Granon et al., 1995; Hasselmo, 1999; Anagnostaras et al., 2003) and has profound effects on the excitability and intrinsic firing patterns of neurons (Krnjević, 1993). One of the most intriguing examples of cholinergic regulation involves the generation of intrinsic persistent firing (Egorov et al., 2002; Fransén et al., 2006; Tahvildari et al., 2008). In the presence of muscarinic acetylcholine receptor agonists a brief depolarizing stimulus can lead to the firing of a neuron for several minutes, far outlasting the original stimulus (Egorov et al., 2002; Sidiropoulou et al., 2009; Rahman and Berger, 2011). Persistent firing is thought to depend on the action of muscarinic agonists to promote the appearance of an excitatory slow afterdepolarization (sADP) following a brief burst of action potentials, in contrast to the brief afterhyperpolarization (AHP) that normally follows a burst of spikes in the absence of cholingergic stimulation (Haj-Dahmane and Andrade, 1998). When suprathreshold, the sADP can result in persistent non-accommodating firing of the neurons.

sADPs have been described in neurons from many areas of the central and peripheral nervous system, including hippocampus (Fraser and MacVicar, 1996; McQuiston and Madison, 1999), nucleus accumbens (D’Ascenzo et al., 2009), prefrontal cortex (Haj-Dahmane and Andrade, 1998; Yan et al., 2009), lateral amygdala (Yamamoto et al., 2007), and olfactory bulb (Constanti et al., 1993). Different molecular mechanisms and neuromodulators have been implicated in the generation of sADPs. In addition to the importance of muscarinic receptor stimulation for sADPs in many cells (Krnjević et al., 1971; Constanti et al., 1993; Fraser and MacVicar, 1996; Haj-Dahmane and Andrade, 1998; McQuiston and Madison, 1999; Egorov et al., 2006; Pressler et al., 2007; Hofmann and Frazier, 2010), sADPs in some neurons have been reported following stimulation of 5-HT_2_ receptors (Araneda and Andrade, 1991; Spain, 1994; Zhang and Arsenault, 2005), α_1_ adrenergic receptors (Araneda and Andrade, 1991), D1 dopamine receptors (Yamamoto et al., 2007; Sidiropoulou et al., 2009), or metabotropic glutamate receptors (Greene et al., 1992, 1994). Pharmacological studies have revealed that a common requirement for sADP generation is the receptor-mediated activation of the phospholipase C (PLC) signaling pathway combined with Ca^2+^ influx into the cell through voltage-gated Ca^2+^ channels (Haj-Dahmane and Andrade, 1998; Pressler et al., 2007; Yan et al., 2009; Hofmann and Frazier, 2010). Such studies have led to the suggestion that the sADP is generated by a calcium-activated nonselective cation (CAN) current (*I_CAN_*). In support of this view, both *I_CAN_* and the sADP require PLC signaling and intracellular Ca^2+^, are enhanced by depolarization, and are blocked by flufenamic acid (FFA; Haj-Dahmane and Andrade, 1998; Pressler et al., 2007; Yamamoto et al., 2007; Yan et al., 2009; Hofmann and Frazier, 2010).

Previous studies have suggested that the CAN current underlying the sADP is carried, at least in part, through the TRPC channel subclass of the transient receptor potential (TRP) channel family (Fowler et al., 2007; Yan et al., 2009; Rahman and Berger, 2011). Similar to *I_CAN_* and the sADP, these nonselective cation channels are activated by PLC-dependent signaling cascades. Although TRPC channels are not directly activated by intracellular Ca^2+^, their opening is enhanced by intracellular Ca^2+^ (Clapham, 2003; Montell, 2005). Unlike many CAN channels, which are selectively permeable to monovalent cations and do not conduct Ca^2+^ (Yellen, 1982; Partridge et al., 1994), TRPC homomeric channels have a high permeability to Ca^2+^ (Okada et al., 1998; Philipp et al., 2000; Schaefer et al., 2000). However, when coexpressed with TRPC1 subunits, the resulting heteromeric TRPC channels exhibit a reduced calcium permeability (Storch et al., 2012), more consistent with the properties of a CAN channel.

A second class of TRP channels that are attractive candidates for *I_CAN_* and the sADP are the TRPM4 and TRPM5 members of the melastatin subfamily of TRP channels, which have numerous critical roles in transporting ions across cell membranes (Clapham, 2003; Montell, 2005; Ramsey et al., 2006). Similar to *I_CAN_*, both TRPM4 and TRPM5 are calcium-activated, monovalent cation-selective channels that require PLC signaling cascades for their activation (Launay et al., 2002; Hofmann et al., 2003; Liu and Liman, 2003; Nilius et al., 2003, 2006; Prawitt et al., 2003). Previous studies have suggested that these channels may contribute to *I_CAN_* in cardiac muscle and other non-neural cells. The clearest role of TRPM5 is in taste transduction as mice with a targeted deletion of TRPM5 have little or no ability to detect physiologically relevant concentrations of bitter or sweet tastants (Zhang et al., 2003; Damak et al., 2006). In addition, based solely on its expression pattern, two studies have suggested that these channels may also contribute to the sADP in respiratory neurons of the pre-Botzinger complex (Mironov, 2008; Mironov and Skorova, 2011). However, the role of TRPM4 and TRPM5 in the central nervous system remains largely unknown.

In this report, we have directly examined the importance of TRPM4 and TRPM5 for the cholinergic-induced sADP in mouse PFC layer 5 pyramidal neurons using both pharmacological and genetic approaches. As most previous studies on the sADP have been carried out in rats, we first confirmed that the carbachol (CCh)-induced sADP is also dependent on intracellular calcium and the PLC pathway in mPFC of mice, the species used for our genetic studies. By examining animals in which TRPM4 and TRPM5 were deleted, either alone or in combination, we found that TRPM5 makes an important contribution to the sADP whereas we could not detect a contribution from TRPM4. As a significant sADP is still observed in mice with combined genetic deletions of both TRPM4 and TRPM5, the sADP must depend on more than one type of ion channel mechanism. Our results are complementary to those of Kim et al. (2013), who found that TRPM4, but not TRPM5, helps generate a depolarization-induced Ca^2+^-dependent slow cation current (DISC) in cerebellar Purkinje neurons.

## Materials and methods

### Brain slice preparation

Coronal brain slices of prefrontal cortex were obtained from 6–7-week-old mice using standard brain slicing methods. Briefly, the animals were killed by cervical dislocation, followed by decapitation and dissection of the brain out of the cranium. The brain was quickly placed in cold modified ACSF (in mM: NaCl 10, Sucrose 195, KCl 2.5, CaCl_2_ 0.5, MgCl_2_ 7, Na_2_PO_4_ 1.25, NaHCO_3_ 25, Glucose 10, Na-pyruvate 2, osmolarity 325 mOsm) for 3–4 min. Brain slices 300–400 μm thick were cut using a Vibratome 3000 (The Vibratome Co., MO) and placed in a beaker containing warm (32°C) standard ACSF for about 30 min (in mM: NaCl 125, KCl 2.5, CaCl_2_ 2, MgCl_2_ 1, Na_2_PO_4_ 1.25, NaHCO_3_ 25, Glucose 25, Na-pyruvate 2, osmolarity 305 mOsm). Slices were then cooled to room temperature for another 30 min before recordings were initiated.

### Electrophysiological recordings

All intracellular recordings were obtained using the whole-cell patch clamp technique using submerged slices continuously superfused with warm ACSF at 34°C. The soma of layer 5 pyramidal neurons were identified and patched after visual approach of the recording pipette using a combination of infrared light and DIC optics. Patch electrodes had resistances of 2–5 MΩ when filled with the following intracellular solution (in mM): K-Gluconate 130, KCl 10, HEPES 10, NaCl 4, EGTA 0.1, MgATP 4, Na_3_GTP 0.3, Na_2_-phosphocreatine 10, osmolarity 305, pH adjusted to 7.25 with KOH. In some experiments, the calcium buffering capacity of the intracellular solution was increased with bis- (*o*-aminophenoxy) -*N*,*N*,N*,N*-tetraacetic acid (BAPTA) and added calcium to bring the free calcium concentration to near 100 nM (Max-chelator).^1^ Recordings were terminated if the series resistance exceeded 20 MΩ for current-clamp recordings. The signals were digitized at 10–50 kHz and low-pass filtered at 1–4 kHz. All recordings were performed in the presence of the following drugs (in μM): carbachol (10), NBQX (10), AP5 (50), picrotoxin (50). When the membrane potential was stable, approximately 10 min after drug application, we induced action potential firing with depolarizing current injections (200 pA for 500 ms) through the patch electrode in current clamp mode to generate sADPs. We measured the peak amplitude of the sADP and its integral over a 10 s period starting at the termination of the current step, which is hereafter called the sADP area. Recordings were analyzed using AXOGRAPH software (Molecular Devices).

### Statistics

Throughout the paper, means are stated as mean ± standard error of the mean (SEM). Student *t*-test and one-way repeated ANOVA measures were used to determine statistical significance. All statistical results were confirmed using the non-parametric Mann-Whitney test. All quantitative analyses were conducted blind to genotype.

### Reagents

NBQX, AP5, and glibenclamide were purchased from Tocris (Ellisville, MO). All other chemicals were purchased from Sigma (St. Louis, MO).

### Histology

Under terminal anesthesia, the mice were trans-cardially perfused with PBS followed by 4% PFA. The brains were then dissected and post-fixed overnight at 4°C in PFA. 50 μm sections were cut on a vibrating slicer and processed using standard immunocytochemical techniques. The following primary antibodies were used: rabbit anti-mouse TRPM5 antibody (1:200; from the laboratory of Charles S. Zuker), goat polyclonal TRPM4 antibody (1:100; SC-27540; Santa Cruz Biotechnology), mouse anti-NeuN antibody (1:100; MAB377B; Millipore). Secondary antibodies used include goat polyclonal anti-rabbit coupled to Alexa Fluor 488, anti-goat coupled to Alexa Fluor 488 and anti-mouse coupled to Rhodamine-Red-X (1:200; purchased from Jackson immunoresearch laboratories). All images were taken using a laser-scanning confocal microscope.

### Mice

In some experiments wild-type C57BL/6 J mice from Jackson laboratories were used, as noted. *Trpm5*^−/−^ mice (Damak et al., 2006) were obtained from the laboratory of Robert F. Margolskee and *Trpm4*^−/−^ mice (Barbet et al., 2008) were obtained from the laboratory of Pierre Launay. We crossed each knockout line with wild-type mice to obtain heterozygotes, which were then crossed to yield *Trpm5*^−/−^ and *Trpm4*^−/−^ mice and their matched wild-type littermates. Mice were genotyped as described in the respective references. Double-knockout mice were generated by intercrossing of *Trpm4*^−/−^ and *Trpm5*^−/−^ mice (Barbet et al., 2008). Single- or double-knockout animals were obtained at the expected Mendelian ratio. Animals were bred and maintained under standard conditions, consistent with NIH guidelines and IACUC approved protocols.

## Results

### Carbachol induced sADP in layer 5 neurons of the mouse mPFC

**Figure 1:**
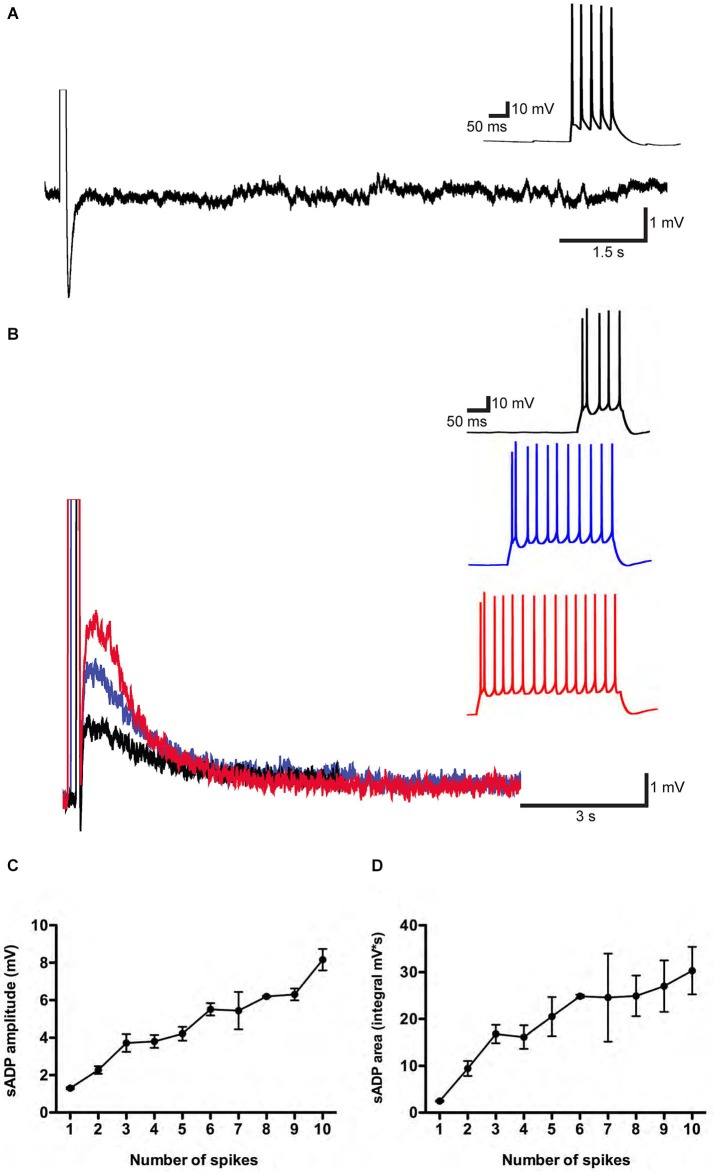
**A muscarinic receptor-dependent slow afterdepolarization (sADP) is observed in PFC layer 5 pyramidal neurons. (A)** In the absence of carbachol (CCh). Representative traces showing an AHP following a burst of action potentials induced by a 200 pA depolarizing current step. Inset shows action potential firing during the current step. Initial resting potential was −66 mV. **(B)** In the presence of 10 μM CCh. Representative traces show sADPs following a burst of action potentials in response to 200 pA depolarizing current steps of increasing duration (black trace: 100 ms; blue trace: 300 ms; red trace: 400 ms). Insets show action potential firing during the depolarization. Initial V_m_ was −65 mV. **(C)** and **(D)** Input-output relations plotting sADP amplitude and area as a function of the number of action potentials elicited by increasing amounts of depolarizing charge injection in the presence of CCh. *n* = 4–8 for each point.

We first examined the conditions in which the sADP could be reliably induced in whole cell current clamp recordings from layer 5 pyramidal neurons from mouse PFC in acute coronal slices, as most previous studies have been performed in rats. Pyramidal neurons were identified based on their morphology and firing properties observed under current-clamp mode (Yang et al., 1996). Muscarinic acetylcholine receptors were activated with 10 μM CCh and ionotropic glutamate and GABA receptors were blocked with 10 μM NBQX, 50 μM AP5 and 50 μM PTX to inhibit fast synaptic transmission. A train of action potentials was elicited in layer 5 pyramidal neurons by the application of 100–500 ms depolarizing current pulses. In the absence of CCh the spike train was followed by a fast AHP (Figure 1A).

After bath application of CCh, we observed a prominent sADP following the evoked spikes (Figure 1B). We quantified the input-output relationship by plotting sADP amplitude and area as a function of spike number elicited by progressively greater amounts of charge injection. Increasing amounts of depolarizing charge elicited a correspondingly greater number of spikes during the depolarizing stimulus, followed by sADPs of increasing amplitude and area (Figures 1C,D). These results are in agreement with previous findings from rats (Haj-Dahmane and Andrade, 1998).

### Calcium influx triggered the sADP in layer 5 neurons

**Figure 2:**
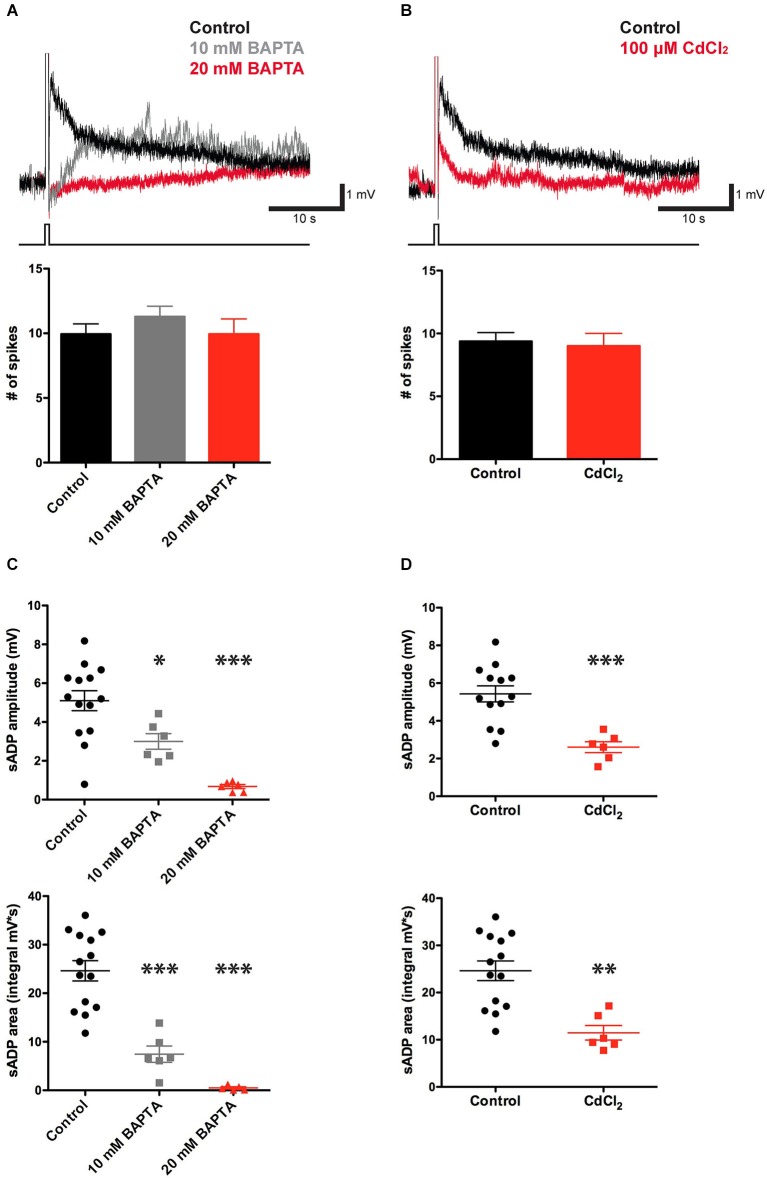
**CCh-induced sADP requires intracellular Ca^2+^. (A)** Applying BAPTA in the patch pipette solution decreased the sADP in presence of 10 μM CCh. Inset: Superimposed traces showing the sADP following action potentials induced by a depolarizing current step (bottom trace) in CCh under control conditions (black trace) or with 10 mM (gray trace) or 20 mM (red trace) BAPTA in the patch pipette solution. Initial V_m_ was −66 mV. Bar graph, number of action potentials during the depolarizing step (200 pA for 500 ms) was not significantly different among groups (*n* = 7–16, *P* = 0.31 and 1 respectively). **(B)** Bath application of Cd^2+^ reduced the sADP. Inset: sADPs induced by a depolarizing current step (in CCh) under control conditions (black trace) or in the presence of 100 μM Cd^2+^ (red trace). Bar graph, number of spikes during the depolarization was not affected by Cd^2+^ (*n* = 6–16, *P* = 0.77). **(C)** BAPTA significantly reduced peak amplitude (*n* = 6–14, * *P* < 0.05, *** *P* < 0.0001) and area (*n* = 6–14, *** *P* < 0.0001) of sADP triggered by a 200 pA depolarizing current step for 500 ms. **(D)** Cd^2+^ decreased sADP amplitude (*n* = 6–13, *** *P* < 0.001) and area (*n* = 6–14, ** *P* < 0.01).

sADPs, induced following spike firing during activation of muscarinic acetylcholine receptors, metabotropic glutamate receptors or other neuromodulators in layer 5 neocortex neurons (Greene et al., 1994; Haj-Dahmane and Andrade, 1998; Yan et al., 2009), layer 2–3 olfactory cortex neurons (Constanti et al., 1993), hippocampal CA1 pyramidal neurons (Fraser and MacVicar, 1996) or lateral amygdala neurons (Yamamoto et al., 2007), have been found to require calcium influx. To determine whether intracellular Ca^2+^ is necessary for the sADPs in our experiments, we included the Ca^2+^ chelator, 1,2-bis(o-aminophenoxy) ethane-*N, N, N’, N’*-tetraacetic acid (BAPTA; 10–20 mM), in the internal solution of the patch pipette. BAPTA produced a substantial reduction in both the peak amplitude and area of the sADP (Figure 2) as previously reported (Haj-Dahmane and Andrade, 1998; Egorov et al., 2006; Yan et al., 2009; Hofmann and Frazier, 2010). The amplitude of the sADP following a suprathreshold stimulus of 100 pC was reduced from 5.10 ± 0.52 mV in control conditions (*n* = 14) to 3.00 ± 0.40 mV (*n* = 6, *P* = 0.02; paired *t*-test) with 10 mM BAPTA in the patch pipette. The sADP was further reduced to only 0.68 ± 0.13 mV (*n* = 6, *P* < 0.0001; paired *t*-test) with 20 mM BAPTA. The sADP integral was also sensitive to the chelators, being reduced from 24.62 ± 2.10 mV·s in control conditions (*n* = 14) to 7.43 ± 1.68 mV·s with 10 mM BAPTA (*n* = 6, *P* < 0.0001; paired *t*-test) and 0.49 ± 0.22 mV·s (*n* = 5, *P* < 0.0001; paired *t*-test) with 20 mM BAPTA.

As spiking is expected to trigger Ca^2+^ influx through voltage-gated Ca^2+^-channels, we tested the effect of CdCl_2_ (100 μM), a general Ca^2+^-channel blocker, on the sADP. As shown in Figures 2B,D, action potential bursts in the presence of Cd^2+^ produced a smaller sADP compared to that seen following a burst with a similar number of spikes under control conditions. Thus, the sADP was reduced from 5.43 ± 0.43 mV in control conditions (*n* = 13) to 2.60 ± 0.29 mV in the presence of Cd^2+^ (*n* = 6, *P* < 0.001; paired *t*-test). Similarly the sADP integral in control conditions, 24.62 ± 2.09 mV·s (*n* = 14), was significantly larger than the integral in the presence of CdCl_2_, 11.70 ± 1.86 mV·s (*n* = 6, *P* = 0.001; paired *t*-test). Thus the sADP in the mouse PFC requires Ca^2+^ influx through voltage-gated Ca^2+^-channels, consistent with previous findings in rats (Pressler et al., 2007).

### The sADP is sensitive to blockers of non-selective cation currents and PLC

**Figure 3:**
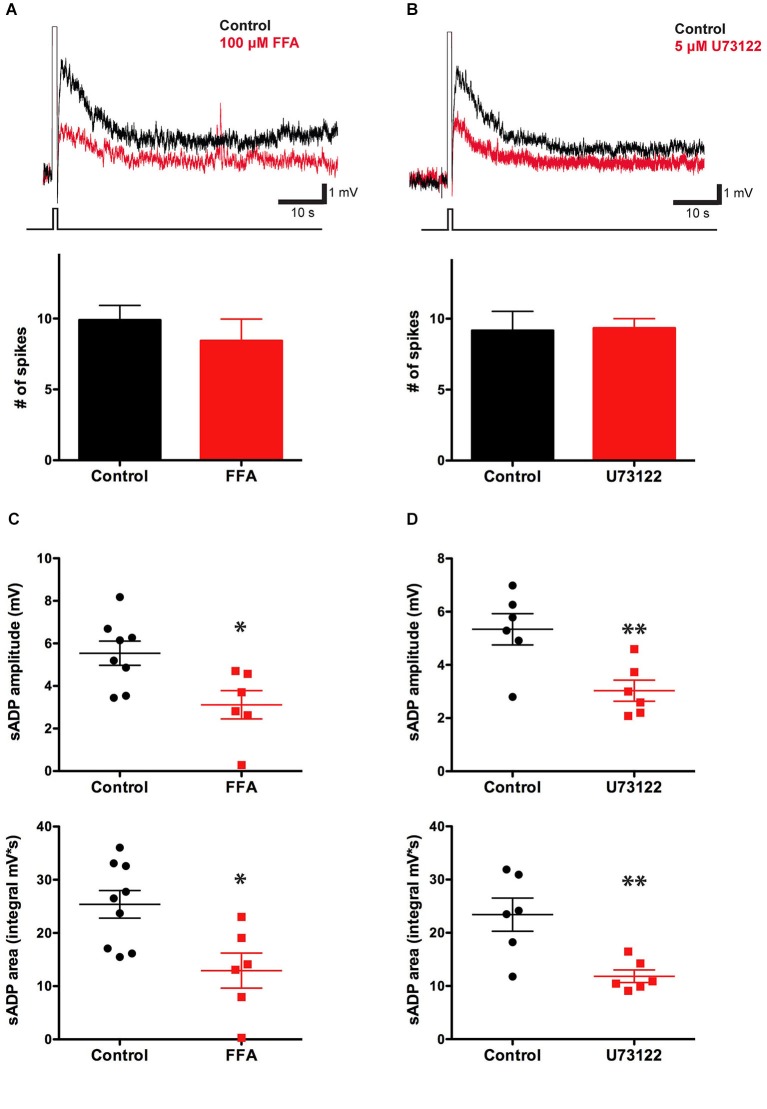
**The sADP is sensitive to a blocker of non-specific cationic currents and requires the PLC pathway. (A)** The CAN current blocking agent FFA decreased the sADP induced by a depolarizing current step (in 10 μM CCh). Inset: sADP under control conditions (black trace) and in presence of FFA (10 μM; red trace). Initial V_m_ was −66 mV. Bar graph, number of spikes during the depolarization (200 pA for 500 ms) was not affected by FFA (*n* = 7–10, *P* = 0.42). **(B)** The PLC blocker U73122 decreased the CCh-induced sADP. Inset: Superimposed traces showing sADP induced by a depolarizing current step (bottom trace) in CCh under control conditions (black trace) or with 5 μM U73122 in bath (red trace). Initial V_m_ was −65 mV. Number of spikes during the depolarization (200 pA for 500 ms) was not affected by U73122 (*n* = 6, *P* = 0.91). **(C)** FFA decreased the sADP amplitude (*n* = 6–8, * *P* < 0.05) and area (*n* = 6–9, * indicates *P* < 0.05). **(D)** The PLC blocker U73122 decreased the sADP amplitude (*n* = 6, ** indicates *P* < 0.01) and area (*n* = 6, ** indicates *P* < 0.01).

Previous studies have shown that in pyramidal neurons of the rat prefrontal cortex the muscarinic receptor-dependent sADP is mediated by *I_CAN_* (Haj-Dahmane and Andrade, 1998; Yan et al., 2009). We therefore tested the effects of the *I*_*CAN*_ blocking agent flufenamic acid (FFA, 10 μM) on the ability of layer 5 PFC pyramidal neurons to generate an sADP. Blockade of *I_CAN_* by FFA produced a significant 40% decrease in the CCh-induced sADP amplitude, from 5.43 ± 0.43 mV (*n* = 13) to 3.12 ± 0.67 mV (*n* = 6, *P* = 0.008; paired-*t*-test), and a 50% decrease in sADP area, from 24.62 ± 2.09 mV·s (*n* = 14) to 12.92 ± 3.30 mV·s (*n* = 6, *P* = 0.007; paired *t*-test) (Figures 3A,C). This result is consistent with previous findings and suggests that *I_CAN_* may be important for the sADP driven by cholinergic muscarinic receptor activation.

To examine whether the effect of muscarinic activation on the sADP in mouse PFC was mediated by the PLC pathway, we tested the action of the PLC blocker U73122 (which is an effective blocker of PLC but may also affect other targets, e.g., Horowitz et al., 2005). In the presence of U73122, both the CCh-induced sADP amplitude and area decreased significantly, from 5.34 ± 0.39 mV (*n* = 6) to 3.03 ± 0.40 mV (*n* = 6, *P* = 0.009) and from 23.42 ± 3.12 mV·s (*n* = 6) to 11.83 ± 1.18 mV·s (*n* = 6, *P* = 0.006), respectively (Figures 3B,D), indicating that the PLC cascade is indeed important in mouse as in rat mPFC (Yan et al., 2009).

### The expression of TRPM4 and TRPM5 in mouse mPFC

**Figure 4:**
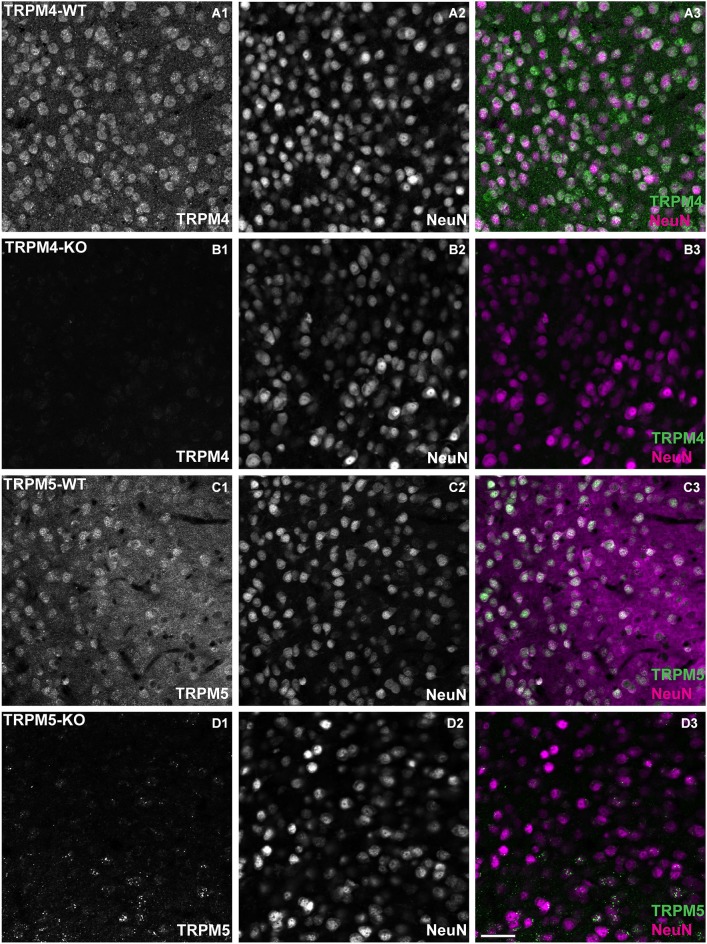
**TRPM4 and TRPM5 expression in coronal slices of adult medial PFC (mPFC).** Immunofluorescence using antibodies directed against TRPM4, TRPM5 and Neuronal Nuclei (NeuN) performed in coronal slices of mPFC. TRPM4 (magenta) and NeuN (green) double/labeled cells were observed in medial prefrontal cortex **(A1–A3)** from TRPM4^+/–^ mice, whereas no TRPM4 immunostaining was observed in sections from *TRPM4*^−/−^ (TRPM4-KO) mice **(B1–B3)**. *TRPM5*^+/–^ heterozygous mice showed TRPM5 immunoreactivity **(C1–C3)**, whereas no TRPM5 immunostaining was observed in sections from *TRPM5*^−/−^ (TRPM5-KO) mice **(D1–D3)**. Staining with TRPM4 and TRPM5 antibody produced predominantly cytosolic labeling and staining of NeuN was primarily localized in the nucleus of the neurons. Scale bars: 50 μm.

As mentioned in the Introduction section, several lines of evidence indicate that TRPM4 and TRPM5 are potential molecular candidates for the generation of *I_CAN_* and the sADP. To examine the relevance of these channels in our experiments, we first characterized the expression of these channels in mPFC. According to the *in situ* hybridization data from the Allen Institute of Brain Science,^2^ TRPM4 and TRPM5 show moderate expression in mouse mPFC. To examine the expression of TRPM4 and TRPM5 protein, we used antibodies directed against TRPM4 (Gerzanich et al., 2009) and TRPM5 (Zhang et al., 2003) in layer 5 cells of prelimbic cortex (Figure 4). To identify neurons, we co-stained with the neuronal marker Neuronal Nuclei (NeuN). TRPM4/NeuN double-labeled (Figures 4A1–A3) and TRPM5/NeuN double-labeled (Figures 4C1–C3) neurons were observed in deep layers of mPFC. The staining signal with antibodies against TRPM4 and TRPM5 was predominantly cytosolic. Moreover the staining represented a specific labeling of the corresponding TRPM channels as the fluorescence signal was absent when we used the TRPM4 and TRPM5 antibodies to label brain slices from mice in which, respectively, TRPM4 and TRPM5 were deleted through homologous recombination (Figures 4B,D).

### Role of TRPM4 and TRPM5 in generating the CCh-induced sADP

**Figure 5:**
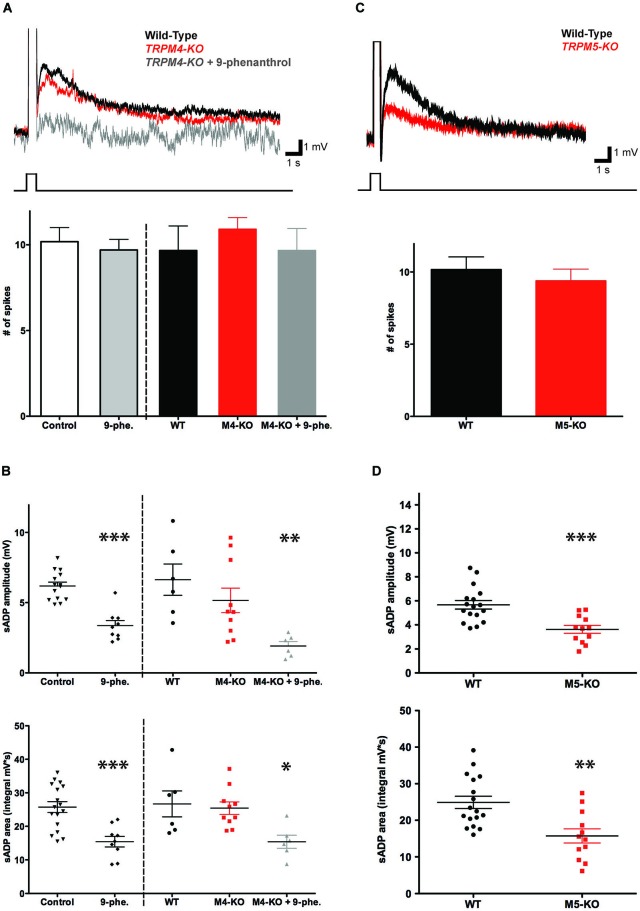
**TRPM5 but not TRPM4 contributes to the CCh-induced sADP. (A)** TRPM4 deletion did not significantly alter the sADP. Inset: superimposed representative traces show sADP induced by a depolarizing current step (200 pA, 500 ms) in CCh for wild-type littermate control mice (black trace), *Trpm4*^−/−^ mice (red trace) and *Trpm4*^−/−^ mice in the presence of 9-phenanthrol (dark gray trace). Initial V_m_ was −66 mV. Bar graph, there was no statistically significant difference in numbers of spikes in wild-type mice under control conditions vs. in the presence of 9-phenanthrol (*n* = 10–22, *P* = 0.71). There were no significant differences in number of spikes among wild-type (WT) mice or *Trpm4*^−/−^ mice (M4-KO) under control conditions or in presence of 9-phenanthrol (M4-KO + 9-phe.); *n* = 6–11, *P* = 0.39 and *P* = 1 respectively. **(B)** Genetic deletion of TRPM4 did not significantly reduce sADP amplitude (top graph; *n* = 6–10, *P* = 0.31) or sADP area (bottom graph; *n* = 6–10, *P* = 0.74). However, application of 9-phenanthrol in both control and *Trpm4*^−/−^ mice decreased significantly peak sADP amplitude (wild-type control vs. 9-phenanthrol, *n* = 9–17, *** *P* < 0.001; wild-type control vs. KO with 9-phenanthrol, *n* = 6, ** *P* < 0.01) and sADP area (in control vs. with 9-phenanthrol, *n* = 9–17, *** *P* < 0.001; in WT vs. KO with 9-phenanthrol, *n* = 6, * *P* < 0.05) by a depolarizing step current (200 pA for 500 ms). **(C)** TRPM5 deletion reduced the sADP. Inset: sADP induced by depolarizing current (200 pA, 500 ms) in CCh in wild-type littermate control mice (black trace) and *Trpm5*^−/−^ KO mice (red trace). Initial V_m_ was −65 mV. Bar graph, TRPM5 deletion (M5-KO) had no effect on number of spikes during the depolarizing step (*n* = 13–18, *P* = 0.54). **(D)** Deletion of TRPM5 significantly decreased sADP amplitude (top graph; *n* = 12–17, *** *P* < 0.001) and area (bottom graph; *n* = 12–17, ** *P* < 0.01).

To determine whether TRPM4 contributes to the burst-triggered sADP generated in the presence of CCh, we used both pharmacological and genetic approaches. We first examined the effect of bath application of two pharmacological agents reported to inhibit TRPM4 but not TRPM5 channels (Grand et al., 2008): glibenclamide and 9-phenanthrol. Glibenclamide (100 μM) had no significant effect on sADP amplitude (6.18 ± 0.28 mV in control conditions (*n* = 14) vs. 5.36 ± 0.24 mV in glibenclamide (*n* = 6, *P* = 0.09; paired *t*-test)) or area (25.75 ± 1.62 mV in control conditions (*n* = 17) vs. 21.29 ± 2.24 mV with glibenclamide (*n* = 9, *P* = 0.16; paired *t*-test)). In contrast 9-phenanthrol (100 μM) did decrease both sADP amplitude (6.18 ± 0.28 mV in control (*n* = 14) vs. 4.86 ± 0.32 mV with 9-phenanthrol (*n* = 9, *P* < 0.0001; paired *t*-test)) and sADP area (25.75 ± 1.62 mV in control conditions (*n* = 17) vs. 15.43 ± 1.58 mV with 9-phenanthrol (*n* = 9, *P* < 0.0001; paired *t*-test)) (Figures 5A,C).

Because the results with the two pharmacological agents were equivocal, we next examined the importance of TRPM4 in generating the sADP using homozygous knockout mice (*Trpm4*^−/−^) (Vennekens et al., 2007). Deletion of TRPM4 had no statistically significant effect on either sADP amplitude or area (Figures 5A,B). Thus, the sADP amplitude and area in the *Trpm4*^−/−^ mice were 5.16 ± 0.87 mV and 25.42 ± 1.87 mV·s (*n* = 10), respectively, compared to 6.64 ± 1.11 mV and 26.69 ± 3.87 mV·s (*n* = 6) in control littermates (*P* = 0.31 for sADP amplitude; *P* = 0.74 for sADP area; paired *t*-test).

How can we reconcile the reduction in the sADP with 9-phenanthrol compared to the lack of effect on the sADP of glibenclamide or TRPM4 deletion? One likely explanation is that the effects of 9-phenanthrol represent an action on some target other than TRPM4 (Wang et al., 1994). To explore this possibility we tested the actions of 9-phenanthrol on the sADP recorded from neurons in slices from the KO mice. Indeed, the compound produced a large reduction in both sADP amplitude (from 6.64 ± 1.11 mV to 1.92 ± 0.31 mV (*n* = 6, *P* = 0.0022; paired *t*-test)) and sADP area (from 26.69 ± 3.87 mV·s to 15.40 ± 1.96 mV·s; *n* = 6, *P* = 0.03; paired *t*-test) (Figures 5A,B). Moreover, these actions were identical to the effect of 9-phenanthrol on the sADP recorded in neurons from slices from wild-type mice. Thus we conclude that the effects 9-phenanthrol were not mediated through blockade of TRPM4 and that this channel makes only a minor contribution, if any, to generating the sADP.

To determine the requirement for TRPM5 in the CCh-induced sADP, we compared the sADP amplitude and area in TRPM5 KO mice (*Trmp5*^−/−^) (Zhang et al., 2003) vs. littermate controls. Genetic deletion of TRPM5 resulted in a statistically significant 40% reduction in the sADP peak amplitude, from 5.67 ± 0.36 mV (*n* = 17) in control to 3.62 ± 0.33 mV in KO mice (*n* = 12, *P* < 0.001; paired *t*-test), and in sADP area, from 24.89 ± 1.68 mV·s (*n* = 17) in control to 15.73 ± 1.94 mV·s in KO mice (*n* = 12, *P* = 0.0014; paired *t*-test) (Figures 5C,D). We also compared other intrinsic cellular properties. We found no difference in resting membrane potential (−69.37 ± 0.64 mV in control (*n* = 14) vs. −70.57 ± 0.53 mV in KO (*n* = 22, *P* = 0.16; paired *t*-test)), membrane input resistance (53.16 ± 3.82 MΩ in control (*n* = 14) vs. 57.09 ± 3.46 MΩ in KO (*n* = 22, *P* = 0.45; paired *t*-test)), and cellular excitability, based on the number of spikes during the depolarization (400 pA for 1 s; 23.87 ± 1.63 spikes in control (*n* = 14) vs. 22.00 ± 1.50 spikes in KO (*n* = 21, *P* = 0.40; paired *t*-test)) (data not shown). Thus, in contrast to TRPM4, we conclude that TRPM5 channels make a significant and selective contribution to generating the sADP.

**Figure 6:**
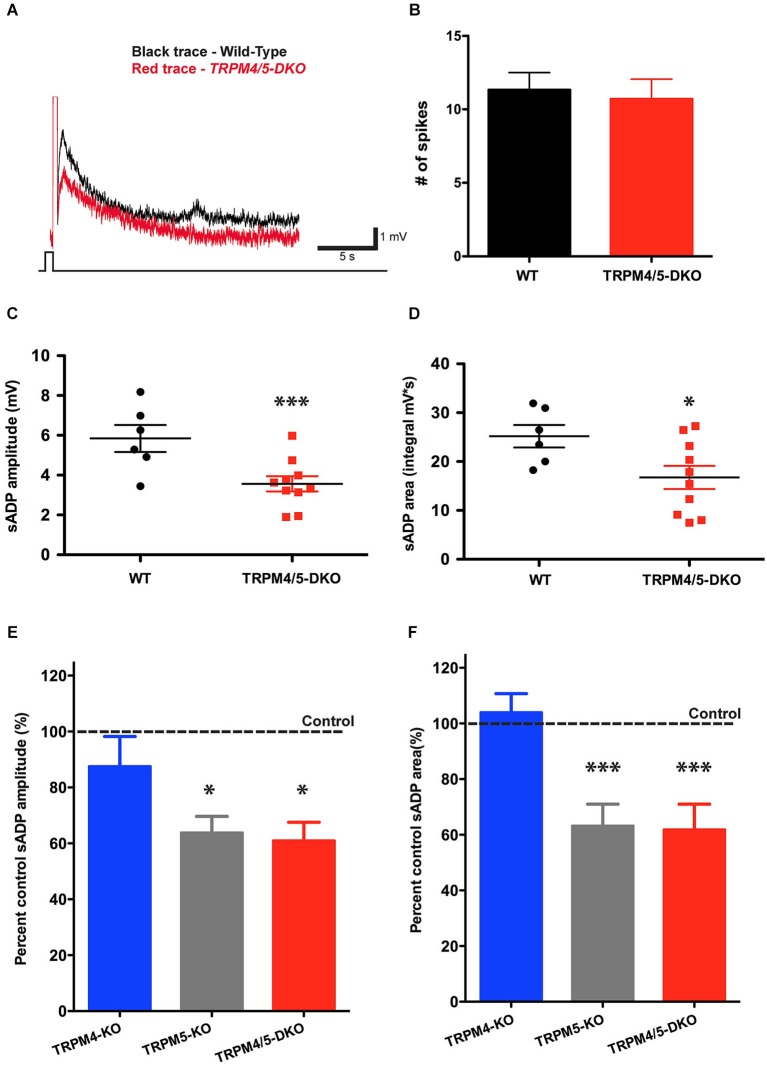
**TRPM4 does not contribute to the residual sADP in TRPM5 KO mice. (A)** Superimposed representative traces show the sADP induced by a depolarizing current step (200 pA, 500 ms) in CCh for wild-type mice (black trace) and *Trpm4/5* double knockout (DKO) mice (red trace). Scale bars, 1 mV and 5 s. Initial V_m_ was −66 mV. **(B)** There is no difference in spike number during current injection between populations of mice (*n* = 6–10, *P* = 0.75). **(C)** *Trpm4/5* -DKO mice generated sADPs with smaller amplitude (*n* = 6–10, ** indicates *P* < 0.01) **(C)** and area (*n* = 6–10, * indicates *P* < 0.05) **(D)** compared to wild-type littermate controls. **(E,F)** Bar graphs showing sADP amplitude **(E)** and area **(F)** in three different genotypes expressed as percentage of sADP in wild-type littermate controls. Normalized sADP amplitude and sADP area show significant differences between *Trpm4*^−/−^ KO vs. *Trpm5*^−/−^ KO and between *Trpm4*^−/−^ KO vs. *Trpm4/5* -DKO mice (one-way ANOVA; *n* = 10–12, * indicates *P* < 0.05 and *** indicates *P* < 0.0001, respectively).

To examine whether TRPM4 might contribute to the residual sADP in the TRPM5 KO mice, we generated double-knockout mice (*Trpm4*^−/−^/*Trpm5*^−/−^), by crossing *Trpm4*^−/−^ mice with *Trpm5*^−/−^ mice (Vennekens et al., 2007). Deletion of both TRPM4 and TRPM5 produced a phenotype nearly identical to that seen in the TRPM5 KO mice (Figure 6). The double KO animals showed a 40% and 33% reduction in sADP peak amplitude and area (Figures 6C,D), respectively, relative to wild-type controls. Thus, the sADP amplitude and area in *Trpm4*^−/−^/*Trpm5*^−/−^ (DKO) mice were 3.57 ± 0.38 mV and 16.74 ± 2.36 mV·s (*n* = 10), compared to 5.85 ± 0.70 mV and 25.18 ± 2.29 mV·s (*n* = 6) in control littermates (*P* < 0.01 for sADP amplitude; *P* < 0.02 for sADP area; paired *t*-test) (Figures 6A–D). We also found no difference in resting membrane potential (−69.01 ± 1.51 mV in control (*n* = 5) vs. −70.24 ± 0.58 mV in the DKO (*n* = 15, *P* = 0.36; paired *t*-test)), membrane input resistance (58.66 ± 3.78 MΩ in control (*n* = 5) vs. 53.39 ± 3.64 MΩ in the DKO (*n* = 15, *P* = 0.40; paired *t*-test)), and cellular excitability (31.40 ± 3.00 spikes in control (*n* = 5) vs. 28.53 ± 1.96 spikes in the DKO mice (*n* = 15, *P* = 0.46; paired *t*-test), elicited by a 400 pA, 1 s depolarizing step). Therefore, our results provide direct evidence that TRPM5, but not TRPM4, channels make an important contribution towards the generation of the sADP in layer 5 pyramidal neurons in mouse mPFC. Moreover TRPM4 is insufficient to maintain the sADP in the absence of TRPM5 (Figures 6E,F).

## Discussion

Previous studies have shown that activation of muscarinic receptors can enable the generation of a calcium-dependent sADP following a brief burst of action potentials in certain cortical neurons (Haj-Dahmane and Andrade, 1998, 1999; Yan et al., 2009). When large enough this afterpotential can support persistent firing (Egorov et al., 2002). In this report, we used a combination of cellular, molecular, electrophysiological and genetic approaches to dissect the molecular mechanisms underlying the muscarinic-receptor-dependent sADP. We have confirmed that activation of ACh receptors with carbachol results in the generation of an sADP in mouse PFC layer 5 pyramidal neurons. Moreover, we confirmed that this sADP depends on activation of a PLC signaling cascade and requires intracellular calcium signaling. In addition, we have provided novel evidence that TRPM5 contributes to the sADP whereas TRPM4 appears less important.

As mentioned earlier, sADPs have been described following activation of a diverse array of G-protein-coupled receptors in many areas of the central and peripheral nervous system. CAN channel activation is thought to serve as the common mechanism to regulate the sADP downstream of these receptors. CAN channels have been identified in a number of neuronal and non-neuronal cell types. In cells where these channels have been most thoroughly characterized, they have been shown to be permeable to monovalent cations, with little permeability to calcium (Yellen, 1982; Partridge et al., 1994). However, in many neurons, including prefrontal cortex neurons, it is less clear as to whether the current underlying the sADP is indeed calcium impermeant. This issue is important in terms of efforts to provide a molecular characterization of the channels underlying *I*_CAN_ and the associated sADP.

There is strong evidence in non-neuronal cells implicating TRPM4 and TRPM5 as CAN channels (Launay et al., 2002; Hofmann et al., 2003; Liu and Liman, 2003; Nilius et al., 2003, 2006; Prawitt et al., 2003; Vennekens et al., 2007; Barbet et al., 2008). When expressed in heterologous cells, recombinant TRPM4 and TRPM5 have been shown to form calcium-activated nonselective cation channels with little or no permeability to calcium (Launay et al., 2002; Hofmann et al., 2003; Liu and Liman, 2003), properties very similar to CAN channels in many native tissues (Yellen, 1982; Partridge et al., 1994). In addition to the requirement for intracellular Ca^2+^, activation of TRPM4 or TRPM5 requires stimulation of G-protein-coupled receptor PLC signaling cascades. In neurons, there is one report supporting the recruitment of TRPM4 in the rhythmic activity of respiratory neurons of the Pre-Botzinger complex based solely on expression patterns (Mironov, 2008). However, we are not aware of any previous direct evidence that these channels underlie any neuronal sADP.

Our results from gene knockout mice suggest that TRPM4 does not significantly contribute to the sADP. This result differs from our finding that 9-phenanthrol, which selectively blocks TRPM4 vs. TRPM5 channels (Grand et al., 2008), produced a significant reduction in the sADP of wild-type mice. However, as we found that that 9-phenanthrol produced a similar reduction in the sADP in *Trpm4*^−/−^ mice, this pharmacological agent must act on some target other than TRPM4 channels. This conclusion is supported by our finding that a different pharmacological agent that targets TRPM4 channels, glibenclamide, had no effect on the sADP in wild-type mice.

In contrast to the lack of involvement of TRPM4 channels in the sADP, we found that TRPM5 channels make an important contribution to the slow depolarization. However, the reduction in the sADP upon deletion of TRPM5 was not complete, with the sADP in the TRPM5 KO mice reduced by approximately 40% relative to the sADP in wild-type mice. The incomplete reduction in the sADP was not a result of compensation by TRPM4 as the combined deletion of both TRPM4 and TRPM5 reduced the sADP by a similar extent (40% decrease) as observed upon deletion of TRPM5 alone.

The above results indicate that additional channels, apart from TRPM4 and TRPM5, must make an important contribution to the sADP. Channels containing TRPC5 (Fowler et al., 2007; Yan et al., 2009) are attractive candidates as TRPC5 channels are also activated by PLC-dependent G protein signaling cascades (Clapham, 2003; Montell, 2005). Although these channels are not directly activated by Ca^2+^, increases in intracellular calcium greatly potentiate G protein-dependent TRPC5 opening. Recent studies indicate that expression of dominant negative TRPC5 channels strongly suppresses the sADP in prefrontal cortex neurons (Yan et al., 2009). Moreover, injection of a C-terminal TRPC5 peptide that contains a PDZ domain binding site, and thus acts as an inhibitor of TRPC5 activation, also decreases the sADP (Zhang et al., 2011). Unlike most CAN channels, and TRPM4 and TRPM5 channels, TRPC5 channels have a high permeability to calcium. However, when TRPC5 subunits assemble with TRPC1 subunits, the resultant heteromeric channels show a lower permeability to Ca^2+^ (Storch et al., 2012), more consistent with the properties of CAN channels. One interesting possibility suggested by the Ca^2+^ permeability of TRPC channels is that TRPM5 channels may act as downstream effectors of TRPC channels during muscarinic activation to enhance the sADP. However, a recent study failed to confirm a role for TRPC5 or TRPC6 in generating the cholinergic afterdepolarization in mouse prefrontal cortex (Dasari et al., 2013).

TRPM4 was recently shown to contribute to the depolarization-induced slow cation current (DISC) in cerebellar Purkinje neurons (Kim et al., 2013). In contrast, DISC measured in TRPM5 KO mice was not significantly different from age-matched wild-type littermates. DISC depends on an autocrine action of dopamine release from the Purkinje neuron dendrites and is thought to contribute to excitation and plasticity in these neurons. In contrast the sADP and CAN current observed in PFC and other brain regions require activation of muscarinic ACh receptors through exogenous release or application of cholinergic agonists. Our results in PFC neurons combined with those of Kim et al. in cerebellar Purkinje neurons indicate that TRPM4 and TRPM5 channels play similar but complementary roles in mediating Ca^2+^-dependent cation currents in distinct types of neurons.

Our study for the first time provides evidence that the TRPM5 channel is an important regulator of the muscarinic ACh receptor-dependent sADP in neurons. Here we propose a model in which TRPM5 functions as a central “hub” to detect the conjoint activation of G-protein receptor signaling and Ca^2+^ influx, thereby converting cholinergic stimulation into profound changes of neuronal excitability. As the sADP has been implicated in persistent firing, this model also provides a mechanism capable of converting subthreshold inputs into intrinsic persistent firing. This form of sustained firing may be involved in higher order cortical functions such as working memory. Thus, it will be of interest in the future to determine the importance of TRPM5 for both persistent activity and working memory.

## Conflict of interest statement

The authors declare that the research was conducted in the absence of any commercial or financial relationships that could be construed as a potential conflict of interest.

## Footnotes

^1^http://maxchelator.stanford.edu/webmaxc/webmaxcS.htm

^2^http://www.brain-map.org/

## References

[B1] Anagnostaras S. G. Murphy G. G. Hamilton S. E. Mitchell S. L. Rahnama N. P. Nathanson N. M. (2003). Selective cognitive dysfunction in acetylcholine M1 muscarinic receptor mutant mice. Nat. Neurosci. 6, 51–58 10.1038/nn992 12483218

[B2] Araneda R. Andrade R. (1991). 5-Hydroxytryptamine2 and 5-hydroxytryptamine 1A receptors mediate opposing responses on membrane excitability in rat association cortex. Neuroscience 40, 399–412 10.1016/0306-4522(91)90128-b 1851255

[B3] Barbet G. Demion M. Moura I. C. Serafini N. Leger T. Vrtovsnik F. (2008). The calcium-activated nonselective cation channel TRPM4 is essential for the migration but not the maturation of dendritic cells. Nat. Immunol. 9, 1148–1156 10.1038/ni.1648 18758465 PMC2956271

[B4] Clapham D. E. (2003). TRP channels as cellular sensors. Nature 426, 517–524 10.1038/nature02196 14654832

[B5] Constanti A. Bagetta G. Libri V. (1993). Persistent muscarinic excitation in guinea-pig olfactory cortex neurons: involvement of a slow post-stimulus afterdepolarizing current. Neuroscience 56, 887–904 10.1016/0306-4522(93)90135-3 8284041

[B6] Damak S. Rong M. Yasumatsu K. Kokrashvili Z. Perez C. A. Shigemura N. (2006). Trpm5 null mice respond to bitter, sweet and umami compounds. Chem. Senses 31, 253–264 10.1093/chemse/bjj027 16436689

[B7] Dasari S. Abramowitz J. Birnbaumer L. Gulledge A. T. (2013). Do canonical transient receptor potential channels mediate cholinergic excitation of cortical pyramidal neurons? Neuroreport 24, 550–554 10.1097/WNR.0b013e3283621344 23652155 PMC4151306

[B8] D’Ascenzo M. Podda M. V. Fellin T. Azzena G. B. Haydon P. Grassi C. (2009). Activation of mGluR5 induces spike afterdepolarization and enhanced excitability in medium spiny neurons of the nucleus accumbens by modulating persistent Na+ currents. J. Physiol. 587, 3233–3250 10.1113/jphysiol.2009.172593 19433572 PMC2727034

[B9] Egorov A. V. Hamam B. N. Fransen E. Hasselmo M. E. Alonso A. A. (2002). Graded persistent activity in entorhinal cortex neurons. Nature 420, 173–178 10.1038/nature01171 12432392

[B10] Egorov A. V. Unsicker K. von bohlen und Halbach O. (2006). Muscarinic control of graded persistent activity in lateral amygdala neurons. Eur. J. Neurosci. 24, 3183–3194 10.1111/j.1460-9568.2006.05200.x 17156379

[B11] Fowler M. A. Sidiropoulou K. Ozkan E. D. Phillips C. W. Cooper D. C. (2007). Corticolimbic expression of TRPC4 and TRPC5 channels in the rodent brain. PLoS One 2:e573 10.1371/journal.pone.0000573 17593972 PMC1892805

[B12] Fransén E. Tahvildari B. Egorov A. V. Hasselmo M. E. Alonso A. A. (2006). Mechanism of graded persistent cellular activity of entorhinal cortex layer v neurons. Neuron 49, 735–746 10.1016/j.neuron.2006.01.036 16504948

[B13] Fraser D. D. MacVicar B. A. (1996). Cholinergic-dependent plateau potential in hippocampal CA1 pyramidal neurons. J. Neurosci. 16, 4113–4128 8753873 10.1523/JNEUROSCI.16-13-04113.1996 PMC6578995

[B14] Gerzanich V. Woo S. K. Vennekens R. Tsymbalyuk O. Ivanova S. Ivanov A. (2009). De novo expression of Trpm4 initiates secondary hemorrhage in spinal cord injury. Nat. Med. 15, 185–191 10.1038/nm.1899 19169264 PMC2730968

[B15] Grand T. Demion M. Norez C. Mettey Y. Launay P. Becq F. (2008). 9-phenanthrol inhibits human TRPM4 but not TRPM5 cationic channels. Br. J. Pharmacol. 153, 1697–1705 10.1038/bjp.2008.38 18297105 PMC2438271

[B16] Granon S. Poucet B. Thinus-Blanc C. Changeux J. P. Vidal C. (1995). Nicotinic and muscarinic receptors in the rat prefrontal cortex: differential roles in working memory, response selection and effortful processing. Psychopharmacology (Berl) 119, 139–144 10.1007/bf02246154 7659760

[B17] Greene C. Schwindt P. Crill W. (1992). Metabotropic receptor mediated afterdepolarization in neocortical neurons. Eur. J. Pharmacol. 226, 279–280 10.1016/0922-4106(92)90073-5 1358660

[B18] Greene C. C. Schwindt P. C. Crill W. E. (1994). Properties and ionic mechanisms of a metabotropic glutamate receptor-mediated slow afterdepolarization in neocortical neurons. J. Neurophysiol. 72, 693–704 7527076 10.1152/jn.1994.72.2.693

[B19] Haj-Dahmane S. Andrade R. (1998). Ionic mechanism of the slow afterdepolarization induced by muscarinic receptor activation in rat prefrontal cortex. J. Neurophysiol. 80, 1197–1210 9744932 10.1152/jn.1998.80.3.1197

[B20] Haj-Dahmane S. Andrade R. (1999). Muscarinic receptors regulate two different calcium-dependent non-selective cation currents in rat prefrontal cortex. Eur. J. Neurosci. 11, 1973–1980 10.1046/j.1460-9568.1999.00612.x 10336666

[B21] Hasselmo M. E. (1999). Neuromodulation: acetylcholine and memory consolidation. Trends Cogn. Sci. 3, 351–359 10.1016/s1364-6613(99)01365-0 10461198

[B22] Hofmann T. Chubanov V. Gudermann T. Montell C. (2003). TRPM5 is a voltage-modulated and Ca(2+)-activated monovalent selective cation channel. Curr. Biol. 13, 1153–1158 10.1016/s0960-9822(03)00431-7 12842017

[B23] Hofmann M. E. Frazier C. J. (2010). Muscarinic receptor activation modulates the excitability of hilar mossy cells through the induction of an afterdepolarization. Brain Res. 1318, 42–51 10.1016/j.brainres.2010.01.011 20079344 PMC2850114

[B24] Horowitz L. F. Hirdes W. Suh B. C. Hilgemann D. W. Mackie K. Hille B. (2005). Phospholipase C in living cells: activation, inhibition, Ca2+ requirement and regulation of M current. J. Gen. Physiol. 126, 243–262 10.1085/jgp.200509309 16129772 PMC2266577

[B25] Kim Y. S. Kang E. Makino Y. Park S. Shin J. H. Song H. (2013). Characterizing the conductance underlying depolarization-induced slow current in cerebellar Purkinje cells. J. Neurophysiol. 109, 1174–1181 10.1152/jn.01168.2011 23197456 PMC3569132

[B26] Krnjević K. (1993). Central cholinergic mechanisms and function. Prog. Brain Res. 98, 285–292 10.1016/s0079-6123(08)62410-3 8248518

[B27] Krnjević K. Pumain R. Renaud L. (1971). The mechanism of excitation by acetylcholine in the cerebral cortex. J. Physiol. 215, 247–268 5579661 10.1113/jphysiol.1971.sp009467 PMC1331876

[B28] Launay P. Fleig A. Perraud A. L. Scharenberg A. M. Penner R. Kinet J. P. (2002). TRPM4 is a Ca2+-activated nonselective cation channel mediating cell membrane depolarization. Cell 109, 397–407 10.1016/s0092-8674(02)00719-5 12015988

[B29] Liu D. Liman E. R. (2003). Intracellular Ca2+ and the phospholipid PIP2 regulate the taste transduction ion channel TRPM5. Proc. Natl. Acad. Sci. U S A 100, 15160–15165 10.1073/pnas.2334159100 14657398 PMC299934

[B30] McQuiston A. R. Madison D. V. (1999). Muscarinic receptor activity induces an afterdepolarization in a subpopulation of hippocampal CA1 interneurons. J. Neurosci. 19, 5703–5710 10407011 10.1523/JNEUROSCI.19-14-05703.1999 PMC6783057

[B31] Mironov S. L. (2008). Metabotropic glutamate receptors activate dendritic calcium waves and TRPM channels which drive rhythmic respiratory patterns in mice. J. Physiol. 586, 2277–2291 10.1113/jphysiol.2007.149021 18308826 PMC2479557

[B32] Mironov S. L. Skorova E. Y. (2011). Stimulation of bursting in pre-Botzinger neurons by Epac through calcium release and modulation of TRPM4 and K-ATP channels. J. Neurochem. 117, 295–308 10.1111/j.1471-4159.2011.07202.x 21281309

[B33] Montell C. (2005). The TRP superfamily of cation channels. Sci. STKE 2005:re3 10.1126/stke.2722005re3 15728426

[B34] Nilius B. Mahieu F. Prenen J. Janssens A. Owsianik G. Vennekens R. (2006). The Ca2+-activated cation channel TRPM4 is regulated by phosphatidylinositol 4,5-biphosphate. EMBO J. 25, 467–478 10.1038/sj.emboj.7600963 16424899 PMC1383543

[B35] Nilius B. Prenen J. Droogmans G. Voets T. Vennekens R. Freichel M. (2003). Voltage dependence of the Ca2+-activated cation channel TRPM4. J. Biol. Chem. 278, 30813–30820 10.1074/jbc.m305127200 12799367

[B53] Okada T. Shimizu S. Wakamori M. Maeda A. Kurosaki T. Takada N. (1998). Molecular cloning and functional characterization of a novel receptor-activated TRP Ca2+ channel from mouse brain. J. Biol. Chem. 273, 10279–10287 10.1074/jbc.273.17.10279 9553080

[B36] Partridge L. D. Muller T. H. Swandulla D. (1994). Calcium-activated non-selective channels in the nervous system. Brain Res. Brain Res. Rev. 19, 319–325 10.1016/0165-0173(94)90017-5 7820135

[B54] Philipp S. Trost C. Warnat J. Rautmann J. Himmerkus N. Schroth G. (2000). TRP4 (CCE1) protein is part of native calcium release-activated Ca2+-like channels in adrenal cells. J. Biol. Chem. 275, 23965–23972 10.1074/jbc.m003408200 10816590

[B37] Prawitt D. Monteilh-Zoller M. K. Brixel L. Spangenberg C. Zabel B. Fleig A. (2003). TRPM5 is a transient Ca2+-activated cation channel responding to rapid changes in [Ca2+]i. Proc. Natl. Acad. Sci. U S A 100, 15166–15171 10.1073/pnas.2334624100 14634208 PMC299937

[B38] Pressler R. T. Inoue T. Strowbridge B. W. (2007). Muscarinic receptor activation modulates granule cell excitability and potentiates inhibition onto mitral cells in the rat olfactory bulb. J. Neurosci. 27, 10969–10981 10.1523/jneurosci.2961-07.2007 17928438 PMC6672850

[B39] Rahman J. Berger T. (2011). Persistent activity in layer 5 pyramidal neurons following cholinergic activation of mouse primary cortices. Eur. J. Neurosci. 34, 22–30 10.1111/j.1460-9568.2011.07736.x 21645136

[B40] Ramsey I. S. Delling M. Clapham D. E. (2006). An introduction to TRP channels. Annu. Rev. Physiol. 68, 619–647 10.1146/annurev.physiol.68.040204.100431 16460286

[B55] Schaefer M. Plant T. D. Obukhov A. G. Hofmann T. Gudermann T. Schultz G. (2000). Receptor-mediated regulation of the nonselective cation channels TRPC4 and TRPC5. J. Biol. Chem. 275, 17517–17526 10.1074/jbc.275.23.17517 10837492

[B41] Sidiropoulou K. Lu F. M. Fowler M. A. Xiao R. Phillips C. Ozkan E. D. (2009). Dopamine modulates an mGluR5-mediated depolarization underlying prefrontal persistent activity. Nat. Neurosci. 12, 190–199 10.1038/nn.2245 19169252 PMC2727588

[B42] Spain W. J. (1994). Serotonin has different effects on two classes of Betz cells from the cat. J. Neurophysiol. 72, 1925–1937 7823109 10.1152/jn.1994.72.4.1925

[B43] Spencer D. G. Jr. Pontecorvo M. J. Heise G. A. (1985). Central cholinergic involvement in working memory: effects of scopolamine on continuous nonmatching and discrimination performance in the rat. Behav. Neurosci. 99, 1049–1065 10.1037//0735-7044.99.6.1049 3843539

[B56] Storch U. Forst A. L. Philipp M. Gudermann T. Mederos y Schnitzler M. (2012). Transient receptor potential channel 1 (TRPC1) reduces calcium permeability in heteromeric channel complexes. J. Biol. Chem. 287, 3530–3540 10.1074/jbc.m111.283218 22157757 PMC3271006

[B44] Tahvildari B. Alonso A. A. Bourque C. W. (2008). Ionic basis of ON and OFF persistent activity in layer III lateral entorhinal cortical principal neurons. J. Neurophysiol. 99, 2006–2011 10.1152/jn.00911.2007 18256167

[B45] Vennekens R. Olausson J. Meissner M. Bloch W. Mathar I. Philipp S. E. (2007). Increased IgE-dependent mast cell activation and anaphylactic responses in mice lacking the calcium-activated nonselective cation channel TRPM4. Nat. Immunol. 8, 312–320 10.1038/ni1441 17293867

[B57] Wang B. H. Ternai B. Polya G. M. (1994). Specific inhibition of cyclic AMP-dependent protein kinase by the antimalarial halofantrine and by related phenanthrenes. Biol. Chem. Hoppe Seyler 375, 527–535 10.1515/bchm3.1994.375.8.527 7811392

[B46] Yamamoto R. Ueta Y. Kato N. (2007). Dopamine induces a slow afterdepolarization in lateral amygdala neurons. J. Neurophysiol. 98, 984–992 10.1152/jn.00204.2007 17553953

[B47] Yan H. D. Villalobos C. Andrade R. (2009). TRPC channels mediate a muscarinic receptor-induced afterdepolarization in cerebral cortex. J. Neurosci. 29, 10038–10046 10.1523/JNEUROSCI.1042-09.2009 19675237 PMC2747319

[B48] Yang C. R. Seamans J. K. Gorelova N. (1996). Electrophysiological and morphological properties of layers V-VI principal pyramidal cells in rat prefrontal cortex in vitro. J. Neurosci. 16, 1904–1921 8774458 10.1523/JNEUROSCI.16-05-01904.1996 PMC6578693

[B49] Yellen G. (1982). Single Ca2+-activated nonselective cation channels in neuroblastoma. Nature 296, 357–359 10.1038/296357a0 6278324

[B50] Zhang Z. W. Arsenault D. (2005). Gain modulation by serotonin in pyramidal neurones of the rat prefrontal cortex. J. Physiol. 566, 379–394 10.1113/jphysiol.2005.086066 15878946 PMC1464765

[B51] Zhang Y. Hoon M. A. Chandrashekar J. Mueller K. L. Cook B. Wu D. (2003). Coding of sweet, bitter and umami tastes: different receptor cells sharing similar signaling pathways. Cell 112, 293–301 10.1016/s0092-8674(03)00071-0 12581520

[B52] Zhang Z. Reboreda A. Alonso A. Barker P. A. Seguela P. (2011). TRPC channels underlie cholinergic plateau potentials and persistent activity in entorhinal cortex. Hippocampus 21, 386–397 10.1002/hipo.20755 20082292

